# Association between serum osmolality and 28-day all-cause mortality in patients with heart failure and reduced ejection fraction: a retrospective cohort study from the MIMIC-IV database

**DOI:** 10.3389/fendo.2024.1397329

**Published:** 2024-07-15

**Authors:** Qi Zou, Jiazheng Li, Pengyang Lin, Jialiang Ma, Zhiliang Wei, Ting Tao, Guodong Han, Shougang Sun

**Affiliations:** Department of Cardiology, Lanzhou University Second Hospital, Lanzhou, China

**Keywords:** serum osmolality, all-cause mortality, heart failure, U-shaped correlation, MIMIC-IV database

## Abstract

**Background:**

Previous studies have not thoroughly explored the impact of serum osmolality levels on early mortality in heart failure and reduced ejection fraction (HFrEF) patients. The purpose of this study was to investigate the relationship between serum osmolality levels and early all-cause mortality in patients with HFrEF.

**Methods:**

The open access MIMIC-IV database was the source of data for our study. We collected demographic data, vital signs, laboratory parameters, and comorbidities of the included patients and divided them into 3 groups based on their initial serum osmolality on admission, with the primary outcome being all-cause mortality within 28 days of admission. Smoothing Spline Fitting Curve, the Kaplan-Meier survival curve, and Threshold effect analysis were used to assess the relationship between serum osmolality and early mortality in HFrEF patients.

**Results:**

A total of 6228 patients (55.31% male) were included. All-cause mortality within 28 days on admission was 18.88% in all patients. After adjusting for confounders, higher serum osmolality levels were independently associated with an increased risk of 28-days all-cause mortality compared with the reference group (Reference group Q2: 290–309 mmol/L, Q4: HR, 1.82 [95% CI 1.19–2.78] P<0.05, Q5: HR, 1.99 [95% CI 1.02–3.91] P<0.05). Smooth spline fitting revealed a U-shaped association between serum osmolality and 28-days all-cause mortality. Further threshold effect analysis results suggested that each unit increase in serum osmolality level was associated with a 2% increase in 28-days all-cause mortality when serum osmolality levels were ≥ 298.8 mmol/L (HR, 1.019 [95% CI 1.012–1.025] P<0.05).

**Conclusion:**

A U-shaped correlation between initial serum osmolality and 28-days all-cause mortality in HFrEF patients was identified, revealing higher osmolality levels significantly increase mortality risk. These results underscore serum osmolality’s critical role in early mortality among HFrEF patients, highlighting the need for further, larger-scale studies for validation.

## Introduction

Heart failure (HF) remains a leading cause of morbidity and mortality worldwide, presenting a substantial burden to healthcare systems (1). Despite advancements in therapeutic strategies, the prognosis for HF patients is often poor, with a high rate of hospital readmission and mortality within the first year following diagnosis (2).

Research indicates that serum osmolality, a crucial indicator of fluid and electrolyte equilibrium, may play an integral role in the pathophysiology of HF (3). In healthy individuals, serum osmolality is rigorously maintained, serving as a determinant of intracellular and extracellular water distribution (4). Elderly patients with HF frequently present with concurrent comorbidities, such as diabetes, hypertension, and renal disease, that can precipitate further perturbation of serum osmolality (5, 6).

Hyponatremia is recognized as a prognostic marker in HF, correlating with increased rates of hospitalization and mortality (7). Nevertheless, the prognostic value of serum osmolality remains insufficiently examined (8). Elucidating the association between serum osmolality and HF outcomes could enhance patient management and risk assessment. While a limited body of research suggests that atypical serum osmolality correlates with adverse HF prognoses, including heightened mortality and readmission rates, the specific relationship to short-term mortality, particularly within the first 28 days post-admission, is less understood (9, 10). This understanding is vital due to the acute nature of HF exacerbations and the imperative of early prognostic determinants in this phase.

Our study endeavors to elucidate the correlation between serum osmolality and 28-days all-cause mortality in heart failure and reduced ejection fraction (HFrEF) patients post-hospital admission. The findings may offer significant insights into the acute-phase prognostic relevance of serum osmolality in HFrEF, potentially informing clinical interventions and enhancing patient outcomes.

## Methods

### Study population

Our study was a retrospective observational analysis. The data was sourced from the Medical Information Mart for Intensive Care IV (MIMIC-IV) database. MIMIC-IV is a large, freely accessible database comprising de-identified health-related data associated with over forty thousand patients who stayed in critical care units of the Beth Israel Deaconess Medical Center between 2001 and 2019 (11). The database includes information such as demographic data, vital signs, laboratory parameters, comorbidities, and more. Author ZQ completed the “Protecting Human Research Participants” online course from the National Institutes of Health, granting them access to the dataset. The use of the MIMIC-IV database for research purposes has been approved by the Institutional Review Boards of the Massachusetts Institute of Technology and the Beth Israel Deaconess Medical Center, with a waiver of informed consent granted. We successfully gathered information on 6228 patients with HFrEF using PostgreSQL Structured Query Language based on International Classification of Diseases (ICD-9 and ICD-10) codes.

### Inclusion and exclusion criteria

The prerequisites for participation were as follows: Patients diagnosed with HFrEF using ICD-9 and ICD-10 disease diagnosis codes in the MIMIC-IV database.

Patients with the following characteristics are excluded: (1) those under the age of 18; (2) those with an admission stay of fewer than 24 h; (3) those with leukemia and myelodysplastic syndrome; (4) those with missing baseline sodium, potassium, urea, and glucose at admission; and (5) data for which the calculated serum osmolality value is anomalous.

### Data collection

Structured Query Language (SQL) for PostgreSQL (version 9.6) was used to extract baseline characteristics including gender, age, comorbidities (hypertension, diabetes mellitus, chronic obstructive pulmonary disease, renal disease, cerebrovascular disease, coronary artery disease) as well as laboratory markers and vitals extracted from the MIMIC-IV database for the first 24 hours after hospitalization. Serum osmolality was calculated using the equation [2 × (Na^+^+ K^+^) + (glucose/18) + (urea/2.8)]. In MIMIC-IV, variables with missing data are common, so missing values were estimated using regression imputation. Variables with more than 20% missingness were removed from the model to avoid bias that might result from directly filling in missing values. All screening variables had less than 20% missing values.

### Variables

Initial serum osmolality on admission was calculated and recorded as a continuous variable for patients in this study. As a dichotomous variable, the all-cause death during the course of 28 days was tracked.

We obtained the ultimate outcome variable in accordance with research and guidelines that have been published. This analysis considered the following covariables as factors to consider: (1) demographic data; (2) vital signs; (3) laboratory parameters; and (4) comorbidities.

As a result, the fully adjusted model was developed using the variables below: (1) continuous variables (obtained at baseline): age; gender; heart rate; systolic blood pressure (SBP); diastolic blood pressure (DBP); respiratory rate; pulse oxygen saturation (SPO_2_); anion gap; bicarbonate; creatinine; glucose; bun; calcium; sodium; potassium; platelet; aspartate transaminase (AST); albumin; urea; chloride; NT-proBNP; Hemoglobin; C-Reactive Protein (CRP); serum osmolality level and (2) categorical variables (obtained at baseline): chronic obstructive pulmonary disease (COPD); hypertension; coronary artery disease (CAD); cerebrovascular disease (CVD); diabetes mellitus; renal disease; atrial fibrillation (AF).

### Statistical analysis

Continuous variables are presented as the mean ± standard deviation or median with interquartile range and were compared with Student’s t-test. Categorical variables are presented as frequencies and percentages, and differences between groups were performed with a Pearson chi-square test or Fisher’s exact test. The Lowess Smoothing technique was used to explore the relationship between osmolarity and mortality. To evaluate the incidence rate of primary outcome events among groups according to different levels of serum osmolality, we used Kaplan-Meier survival analysis, and discrepancies among groups were evaluated with log-rank tests. We used Cox proportional hazards models to estimate the hazard ratio (HR) and 95% confidence interval (CI) between serum osmolality and 28-days all-cause mortality, adjusted for multiple models. To avoid overfitting the model because of multicollinearity among variables, we also calculated the variance inflation factor (VIF). Variables with VIF ≥ 5 were excluded. Finally, clinically relevant and prognosis-associated variables were enrolled in the multivariate model: model 1: unadjusted; model 2: adjusted for age and sex; model 3: adjusted for age, sex, heart rate, SBP, DBP, respiratory rate, SPO2, anion gap, bicarbonate, creatinine, glucose, bun, calcium, sodium, potassium, platelet, AST, albumin, urea, chloride, NT-proBNP, hemoglobin, CRP, hypertension, diabetes mellitus, CAD, COPD, renal disease, CVD. Furthermore, a segmented regression model and logarithmic likelihood ratio test were used to analyze the threshold effect between serum osmolality levels and 28-days all-cause mortality.

We used R 4.1.3 (R Foundation for Statistical Computing, Vienna, Austria) and Stata 12.0 (Stata Corporation LLC, College Station, USA) for data analysis. A two-sided P-value < 0.05 was considered statistically significant for all analyses.

## Results

**Figure 1:**
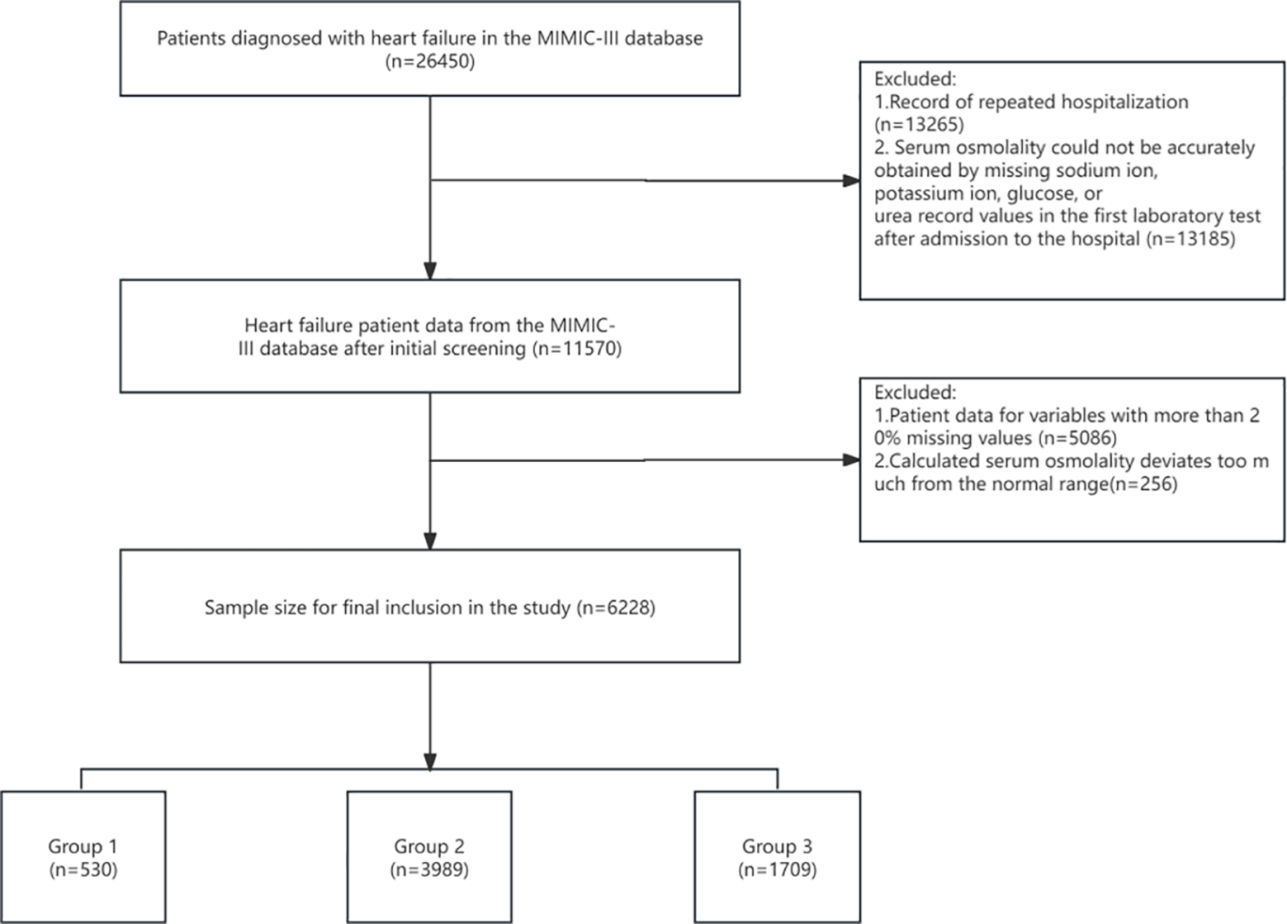
Flowchart of patient selection.

A total of 6228 patients were included in this study, the mean age of the patients was 72.62 ± 13.44 and 3445 (55.31%) were male (**Figure 1**). The mean serum osmolality of all included patients was 304.271 ± 10.87. The all-cause mortality rate within 28 days of admission was 18.88% (**Table 1**).

**Table 1:** Baseline characteristics (N = 6228).

Variable	Serum osmolality(mmoL/L)				P-value
	Total (N=6228)	G1 (<290 mmol/L) (N=530)	G2 (290-310 mmol/L) (N=3989)	G3 (>310 mmol/L) (N=1709)	
Baseline variables					
Age	72.62 ± 13.44	70.40 ± 13.79	71.63 ± 13.68	75.62 ± 12.24	<0.001
Gender					0.009
Male	3445 (55.31%)	288 (54.34%)	2158 (54.10%)	999 (58.46%)	
Female	2783 (44.69%)	242 (45.66%)	1831 (45.90%)	710 (41.54%)	
Vital signs					
Heart rate (bpm)	86.25 ± 18.32	88.35 ± 18.64	86.52 ± 18.09	84.96 ± 18.70	<0.001
SBP (mmHg)	119.53 ± 22.52	117.65 ± 21.93	119.09 ± 22.43	121.14 ± 22.84	0.001
DBP (mmHg)	64.38 ± 15.48	64.27 ± 15.18	64.59 ± 15.34	63.94 ± 15.90	0.307
Respiratory rate (bpm)	19.34 ± 5.23	19.30 ± 5.06	19.15 ± 5.24	19.78 ± 5.23	<0.001
SPO2 (%)	96.86 ± 2.88	96.81 ± 2.79	96.88 ± 2.87	96.82 ± 2.94	0.679
Laboratory parameters					
Anion gap (mmol/L)	14.34 ± 3.30	13.77 ± 3.31	14.03 ± 3.14	15.26 ± 3.50	<0.001
Bicarbonate (mmol/L)	25.47 ± 4.71	25.17 ± 4.22	25.61 ± 4.42	25.23 ± 5.43	0.003
Creatinine (mEq/L)	1.21 ± 0.53	0.98 ± 0.45	1.12 ± 0.47	1.50 ± 0.57	<0.001
Glucose (mg/dL)	7.52 ± 3.45	6.40 ± 1.91	7.14 ± 2.78	8.75 ± 4.69	<0.001
BUN (mg/dL)	30.85 ± 19.38	18.93 ± 10.46	25.00 ± 13.14	48.21 ± 22.51	<0.001
Calcium (mmol/L)	8.61 ± 0.59	8.45 ± 0.57	8.62 ± 0.58	8.64 ± 0.62	<0.001
Sodium (mmol/L)	138.65 ± 4.30	132.02 ± 2.89	138.24 ± 3.25	141.66 ± 4.13	<0.001
Potassium (mmol/L)	4.22 ± 0.66	4.16 ± 0.63	4.17 ± 0.60	4.35 ± 0.76	<0.001
Platelet (10^9/L)	208.31 ± 83.64	212.55 ± 92.05	210.27 ± 82.63	202.41 ± 83.00	0.001
AST (IU/L)	36.14 ± 19.39	37.16 ± 20.01	35.96 ± 19.30	36.25 ± 19.39	0.187
Albumin (g/L)	28.44 ± 17.27	27.73 ± 16.25	28.45 ± 17.44	28.63 ± 17.19	0.487
Urea (mg/dL)	28.15 ± 14.55	18.93 ± 10.46	24.55 ± 11.96	39.41 ± 14.84	<0.001
Chloride (mmol/L)	101.67 ± 5.74	96.41 ± 5.09	101.52 ± 5.10	103.65 ± 6.22	<0.001
NT-proBNP (pg/mL)	7802.32 ± 8079.70	6503.74 ± 6857.10	6926.01 ± 7440.60	10250.43 ± 9266.06	<0.001
Hemoglobin (g/dl)	11.69 ± 2.27	11.67 ± 2.25	11.86 ± 2.25	11.27 ± 2.25	<0.001
C-Reactive Protein (mg/L)	61.79 ± 73.17	60.74 ± 63.50	59.38 ± 71.12	67.60 ± 80.47	0.267
serum osmolality (mmol/L)	304.271 ± 10.87	285.51 ± 3.65	300.89 ± 5.28	317.98 ± 6.09	<0.001
Comorbidity					
COPD	252 (4.05%)	23 (4.34%)	148 (3.71%)	81 (4.74%)	0.183
Hypertension	2833 (45.49%)	292 (55.09%)	1940 (48.63%)	601 (35.17%)	<0.001
CAD	3419 (54.90%)	273 (51.51%)	2194 (55.00%)	952 (55.71%)	0.232
CVD	318 (5.11%)	28 (5.28%)	196 (4.91%)	94 (5.50%)	0.642
Renal disease	762 (12.24%)	61 (11.51%)	409 (10.25%)	292 (17.09%)	<0.001
Diabetes mellitus	2490 (39.98%)	138 (26.04%)	1479 (37.08%)	873 (51.08%)	<0.001
Atrial fibrillation	3163 (50.79%)	265 (50.00%)	1974 (49.49%)	924 (54.07%)	0.006
Events					
28-days all-cause mortality	1176 (18.88%)	97 (18.30%)	596 (14.94%)	483 (28.26%)	<0.001

### Baseline characteristics

**Table 1** lists the baseline characteristics of the study patients according to serum osmolality tertiles. Patients were categorized into three groups (G1: <290 mmol/L, G2: 290–310 mmol/L, G3: >310 mmol/L) based on admission serum osmolality level. The mean levels of serum osmolality in the three groups were G1: 285.51 ± 3.65, G2: 300.89 ± 5.28, G3: 317.98 ± 6.09. The patients in the group with higher serum osmolality were older, predominantly male, and had higher levels of SBP, respiratory rate, bun, creatinine, calcium, sodium, urea, chloride levels, and comorbidities with renal disease and diabetes mellitus were relatively more common. As serum osmolality increased, heart rate, DBP, and platelets showed a tendency to decrease, and comorbidities with hypertension were less common. 28-days all-cause mortality showed a significant incremental trend between serum osmolality groups. Overall, the 28-days all-cause mortality rate in the study population was 18.88%, and the difference in mortality rates between the three groups was statistically significant (18.30% vs. 14.94% vs. 28.26%, P < 0.001), suggesting that increased serum osmolality is associated with a higher risk of 28-days all-cause mortality.

### Smoothing spline fitting curve

**Figure 2:**
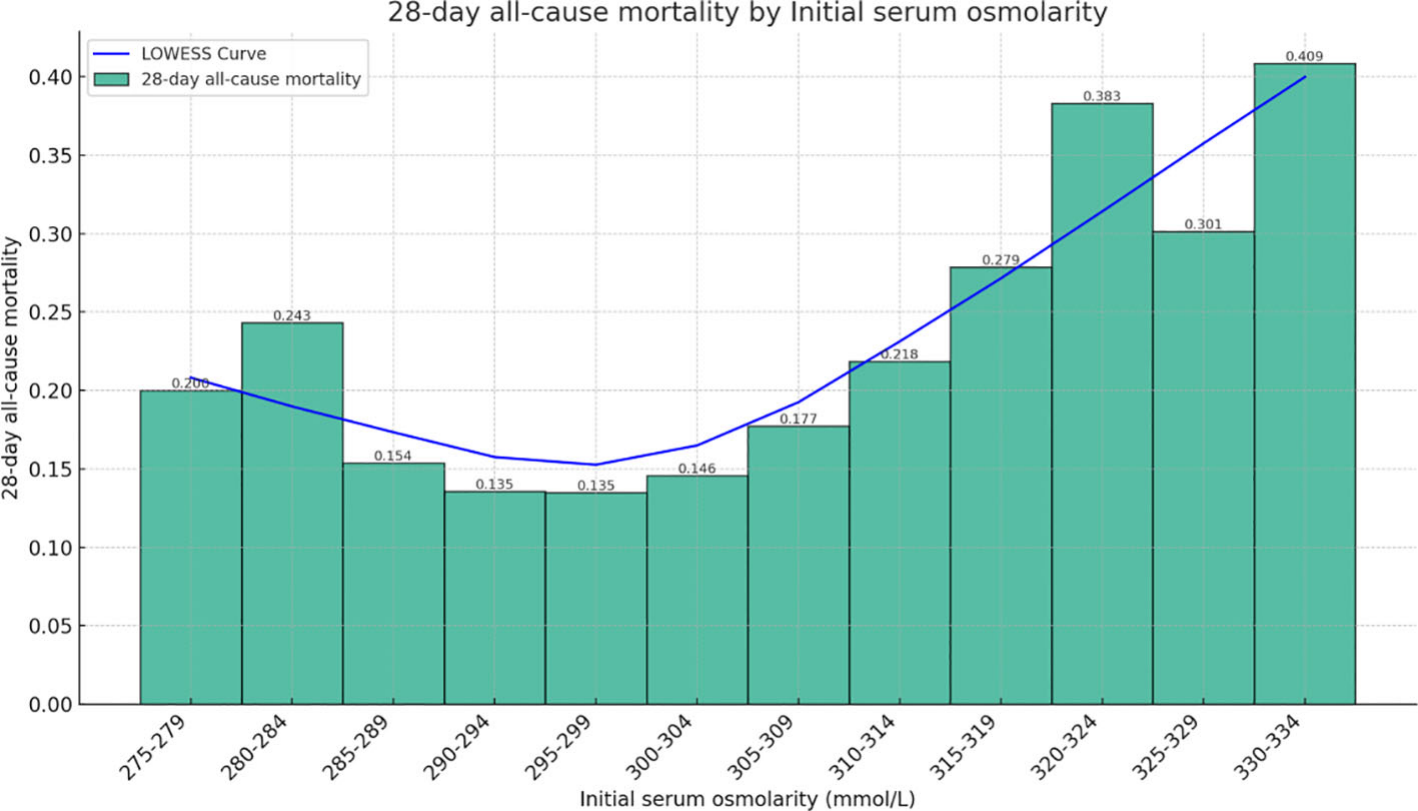
Relationship between serum osmolality and 28-days all-cause mortality in patients with HFrEF. The graph shows a nonlinear relationship.

**Figure 2** shows the relationship between initial serum osmolality on admission and 28-days all-cause mortality among HFrEF patients as determined using the Lowess Smoothing technique. The model produced a nonlinear relationship, with the lowest mortality rates at serum osmolality of approximately 290–299 mmoL/L. For our included patients, the results of Lowess Smoothing showed a U-shaped relationship between initial serum osmolality on admission and 28-days all-cause mortality (**Figure 2**).

### Association between initial serum osmolality and 28-days all-cause mortality in HFrEF

The independent correlation between initial serum osmolality level on admission and 28-days all-cause mortality in HFrEF patients is shown in **Table 2**. When serum osmolality was treated as a continuous variable, it was positively associated with 28-days all-cause mortality. In unadjusted Model I, each unit increase in serum osmolality level was associated with a 3% increase in the risk of 28-days all-cause mortality (HR, 1.03[95% CI 1.03–1.04] P<0.05). In Model II (adjusted for age and sex), the results remained significant (HR, 1.03[95% CI 1.02–1.03] P<0.05). In model III (adjusted for age, sex, and potential confounders), the results did not show significant changes (HR, 1.04[95% CI 1.02–1.06] P<0.05). To further explore the effect of serum osmolality, patients were divided into 5 groups according to the value of serum osmolality (see **Table 2** for details), where the Q2 group (290–309 mmol/L) was used as the reference group, and the results of model I showed that higher initial serum osmolality on admission of the patients was associated with an increase in the 28-days all-cause mortality of the patients after admission, with a gradual increase in the HR starting from Q3 (HR, 1.83[95% CI 1.56 - 2.14] P<0.001) to Q5 (HR, 3.93[95% CI 2.58 - 6.00] P<0.001), and the overall trend in adjusted model II and model III showed consistency with model I.

**Table 2:** Association between serum osmolality and 28-days all-cause mortality in patients with HFrEF.

Variables	Model I (HR,95% CI)	Model II (HR,95% CI)	Model III (HR,95% CI)
Serum osmolality	1.03(1.03, 1.04)*	1.03(1.02, 1.03)*	1.04(1.02, 1.06)*
Serum osmolality quintiles			
Q1 (≤289mmol/L)	1.28(1.01, 1.62)*	1.34 (1.05, 1.71)*	0.88 (0.30, 2.63)*
Q2 (290- 309mmol/L)	Ref.	Ref.	Ref.
Q3 (210- 319mmol/L)	1.83 (1.56, 2.14)*	1.64 (1.39, 1.93)*	1.61 (0.74, 3,52)*
Q4 (320- 329mmol/L)	3.14 (2.55, 3.87)*	2.86 (2.31, 3.54)*	1.62 (0.40, 5.76)*
Q5 (≥330mmol/L)	3.93 (2.58, 6.00)*	3.84 (2.49, 5.92)*	1.79 (0.25, 12.70)*
P value for trend	<0.001	<0.001	<0.001

HR, hazard ratio; CI, confidence interval; Ref, reference; *P value <0.05.

Model I: adjusted for none.

Model II: adjust for age; sex.

Model III: adjusted for age, sex, heart rate, SBP, DBP, respiratory rate, SPO2, anion gap, bicarbonate, creatinine, glucose, bun, calcium, sodium, potassium, platelet, AST, albumin, urea, chloride, NT-proBNP, hemoglobin, CRP, hypertension, diabetes mellitus, CAD, COPD, renal disease, CVD, AF.

### Survival analysis

**Figure 3:**
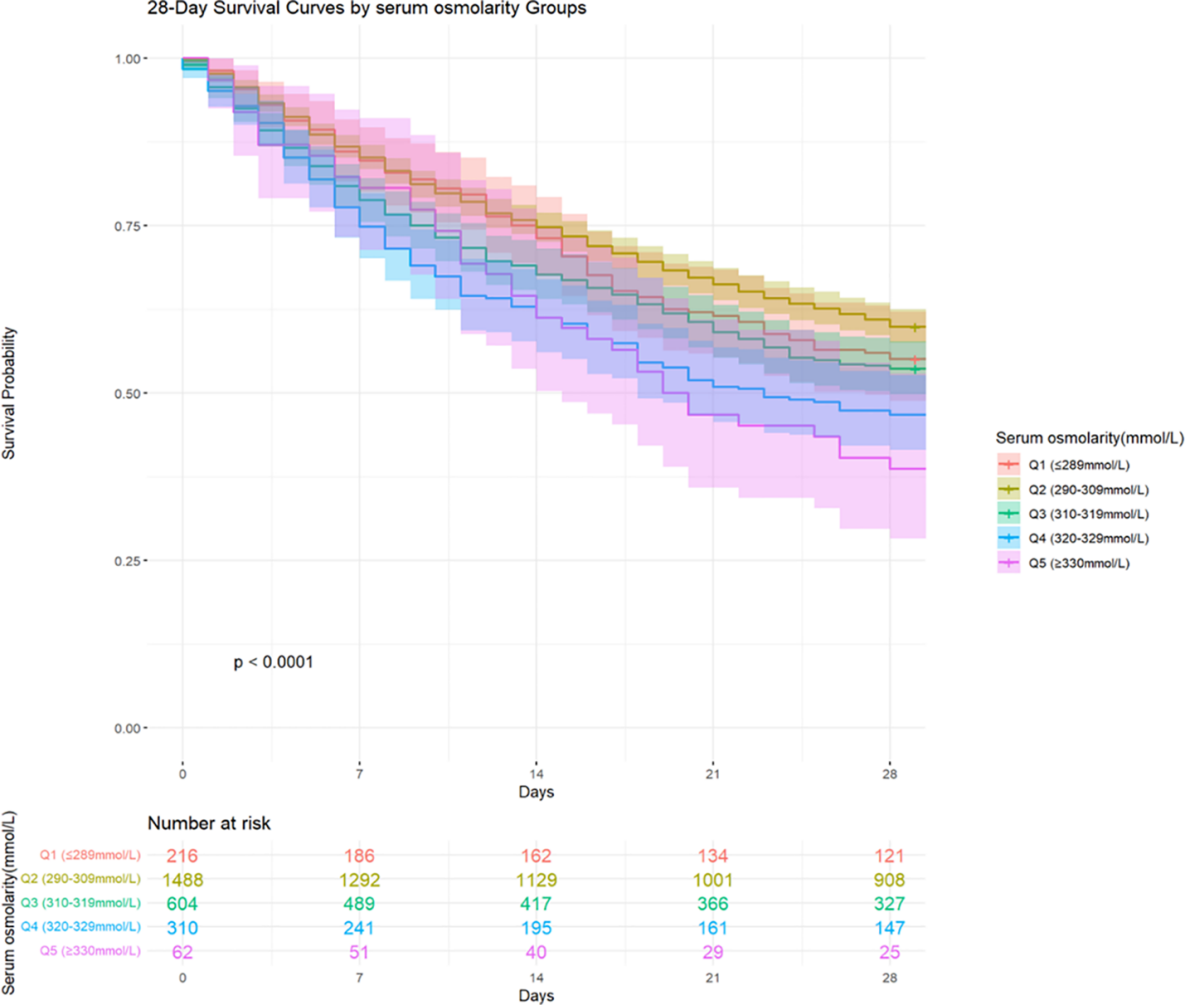
Kaplan–Meier survival analysis curves for 28-days all-cause mortality.

Among the 6228 patients included in the study, 1176 (18.88%) died within 28 days of hospital admission. The 28-days all-cause mortality for Q1 (≤ 289 mmol/L), Q2 (290–309 mmol/L), Q3 (310–319 mmol/L), Q4 (320–329 mmol/L), and Q5 (≥ 330 mmol/L) were 18.30%, 14.94%, 24.31%, 39.86%, and 40.86%, respectively. Kaplan-Meier curves were constructed to visualize the association between serum osmolality quintiles and the 28-days all-cause mortality (**Figure 3**). During the short-term follow-up of 28 days, a statistically significant difference in mortality rates was observed between the groups. Patients had a higher short-term survival rate when serum osmolality was between 290–309 mmol/L, a lower survival rate within 1–14 days of admission when serum osmolality was between 320–329mmol/L, and a lower survival rate within 15–28 days of admission when serum osmolality was above 330mmol/L.

### Threshold effect analysis for the relationship between serum osmolality levels and 28-days all-cause mortality

**Figure 4:**
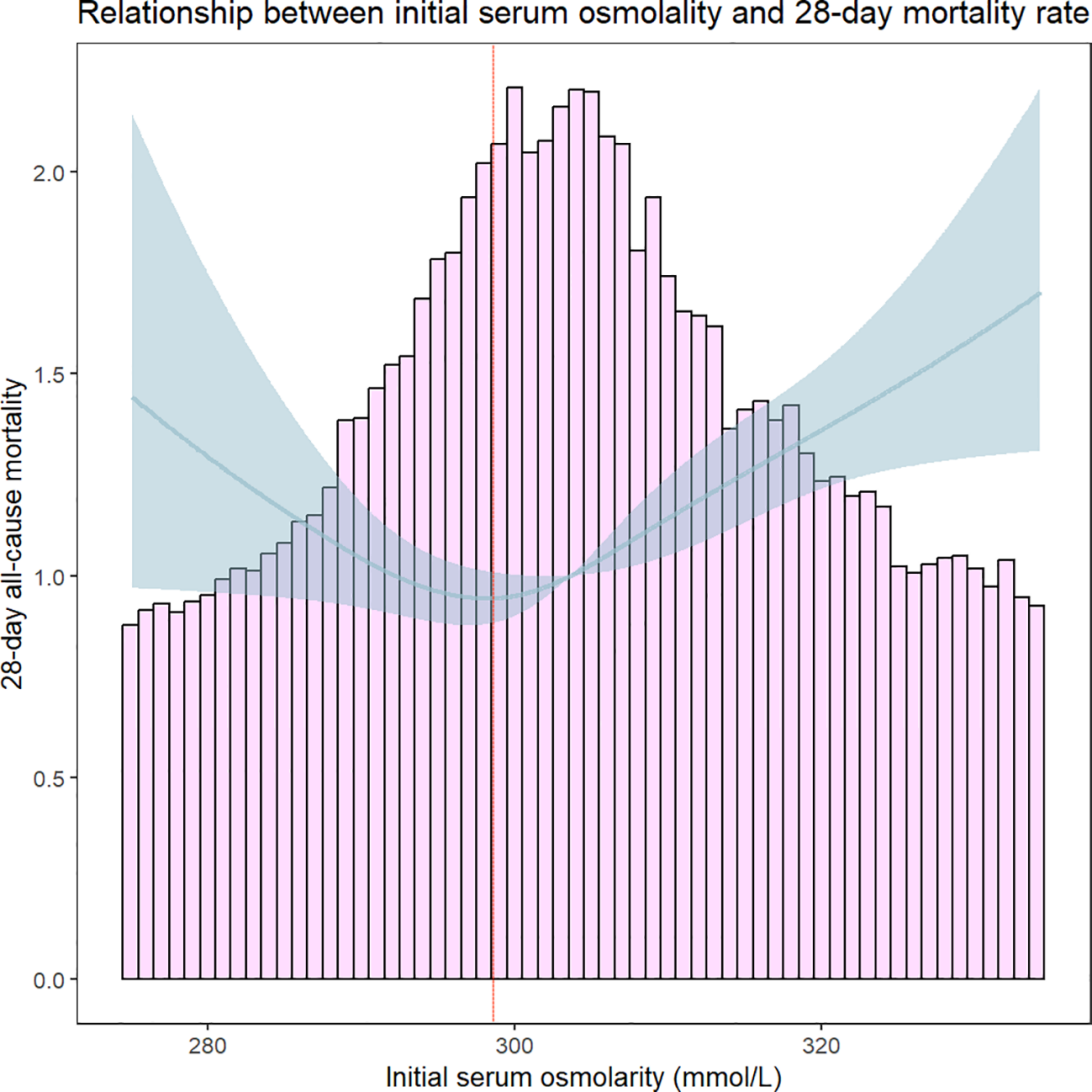
Threshold effect analysis of serum osmolality on 28-day all-cause mortality in Heart Failure Patients. The red dashed line indicates that 28-day all-cause mortality was lowest in HFrEF patients when serum osmolality was 298.8 mmol/L.

Through Lowess Smoothing analysis, it was found that the relationship between serum osmolality levels and 28-days all-cause mortality is U-shaped. Further analysis using a segmented regression model of serum osmolality levels and 28-days all-cause mortality identified a turning point at 298.8 mmol/L of serum osmolality. At a serum osmolality level of 298.8 mmol/L, the mortality rate for patients with HFrEF was the lowest (**Figure 4**). The results suggest that serum osmolality levels are associated with a doubled risk of 28-days all-cause mortality in patients with HFrEF after admission. For serum osmolality levels ≥298.8 mmol/L, each unit increase in serum osmolality level is associated with a 2% increase in mortality within 28 days of admission (HR, 1.019[95% CI 1.012–1.025]). The LRT test was significant (P = 0.002), indicating a non-linear association between serum osmolality levels and 28-days all-cause mortality (**Table 3**).

**Table 3:** Association between serum osmolality and 28-days all-cause mortality in patients with HFrEF.

Models	HR (95%CI)	P-value
Model I		
Fitting model by standard linear regression	1.011(1.006-1.016)	<0.001
Model II		
Fitting model by two-piecewise linear regression		
Turning point (K1)		
<298.833	0.987(0.972-1.002)	0.094
>298.833	1.019(1.012-1.025)	<0.001
LRT test	0.002	

Data were presented as HR (95% CI) P-value; Model I, linear analysis; Model II, non-linear analysis. LRT test, Logarithmic likelihood ratio test (p < 0.05 means Model II is significantly different from Model I, which indicates a non-linear relationship).

## Discussion

To the best of our knowledge, this is the most comprehensive study utilizing the MIMIC-IV database to examine the link between serum osmolality imbalance and mortality among HFrEF patients. Our results reveal that elevated serum osmolality correlates with increased 28-days all-cause mortality in a U-shaped relationship, with the association persisting after adjusting for potential confounders.

**Figure 5:**
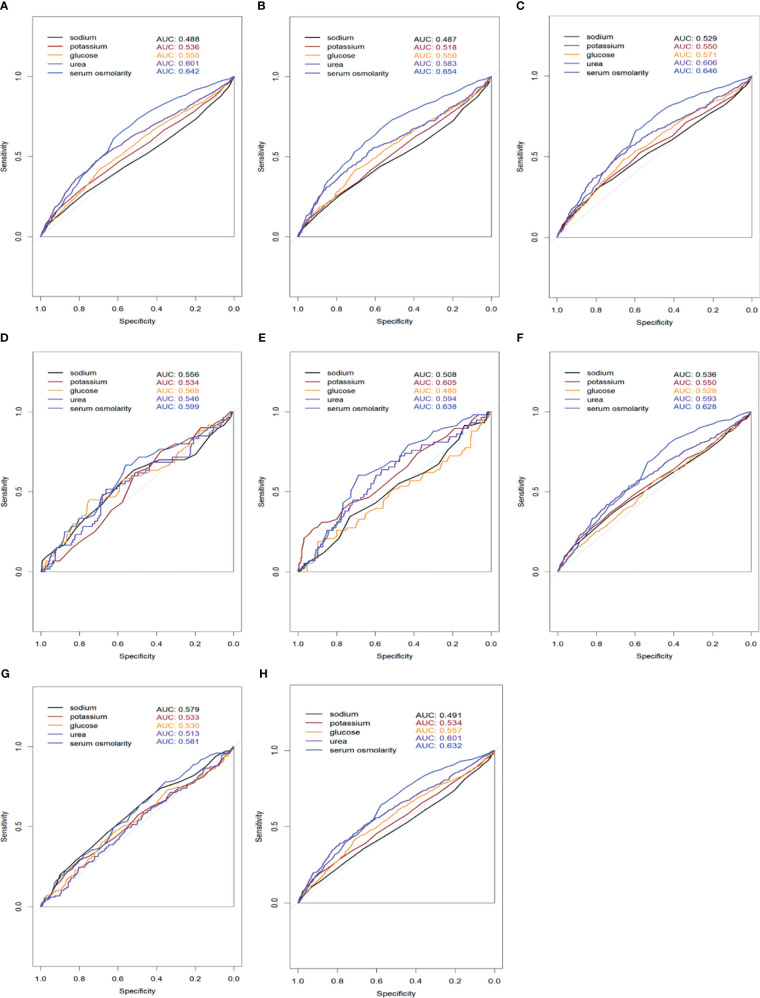
Receiver Operating Characteristic (ROC) curves comparing the sensitivity and specificity of serum osmolality and individual markers (sodium, potassium, glucose, urea) for predicting 28-days all-cause mortality in HFrEF patients. The Area Under the Curve (AUC) values for each marker are displayed in the legend. **(A)** All patients included in the study; **(B)** Patients with coexisting hypertension; **(C)** Patients with coexisting CAD; **(D)** Patients with coexisting CVD; **(E)** Patients with coexisting COPD; **(F)** Patients with coexisting diabetes mellitus; **(G)** Patients with coexisting renal disease; **(H)** Patients with coexisting AF.

Our analysis indicates that individuals with higher serum osmolality are typically older, possess more comorbidities, particularly diabetes and renal disease, and exhibit increased levels of glucose, urea, creatinine, and potassium. These findings align with those of José et al. Previous research has questioned the impact of serum osmolality variations on the short-term prognosis of older HFrEF patients, noting that the confluence of multiple underlying diseases and diverse laboratory index alterations exerts a more pronounced effect on short-term mortality, complicating the interpretation of serum osmolality’s impact (8). To mitigate this complexity, we employed several models: Model I with no adjustments, Model II adjusted for age and gender, and Model III, which further adjusted for multiple confounders. The findings affirm that serum osmolality exerts a direct effect on 28-days all-cause mortality in HFrEF patients, independent of age, sex, comorbidities, and various laboratory measures. Having established the non-linear relationship between initial serum osmolality and 28-days all-cause mortality, we employed threshold effect modeling to corroborate the association. We observed that serum osmolality above 298.8 mmol/L significantly increases 28-days all-cause mortality, reinforcing the non-linear correlation with mortality. To further compare the sensitivity and specificity of serum osmolality in predicting short-term mortality in patients with HFrEF, we analyzed it using ROC curves to describe its AUC value and further compared it with the individual indices (Na+, K+, glucose, urea) used to calculate serum osmolality. In our study, we found that the AUC values of serum osmolality were greater than those of the individual markers (**Figure 5**), demonstrating superior sensitivity and specificity for predicting the short-term prognosis of patients with heart failure. This finding reinforces the need to monitor changes in serum osmolality in patients with severe HFrEF.

Previous research has consistently reported hyponatremia as the most prevalent electrolyte imbalance in hospitalized HF patients, typically precipitated by neurohormonal activation or pharmacological interventions (12). In HF patients, chronic activation of the renin-angiotensin-aldosterone system and the sympathetic nervous system in response to insufficient tissue perfusion promotes water and sodium retention. Moreover, arginine vasopressin, released due to low cardiac output, augments intravascular volume, contributing to hyponatremia development in HF patients (13). Numerous studies have confirmed the association of hyponatremia with increased morbidity and mortality in hospitalized HF patients (14–16). Peng et al. also identified a U-shaped relationship between serum sodium levels and all-cause mortality in HF patients (17). Serum osmolality, a more comprehensive measure, is influenced not only by sodium but also by glucose, urea, and potassium concentrations, which collectively regulate it within a narrow range of 275–295 mmoL/L (18). In our study, the higher osmolality group exhibited elevated glucose, urea, and potassium levels. The influence of serum osmolality on HFrEF patients has been a longstanding concern, with other studies highlighting low osmolality as a significant risk factor for increased morbidity and mortality. José et al. documented that high serum osmolality forecasts poorer outcomes in patients with decompensated HF over one year. However, their study did not elaborate on the impact of confounding factors (8).

Our investigation, focusing on short-term prognosis post-hospital admission (28 days), concurs with these observations, further noting that higher serum osmolality is linked to increased short-term mortality, even after adjusting for confounders using various observational models. Alongside existing literature, the detrimental effects of serum osmolality on HF patients can be rationalized through several mechanisms. Firstly, antidiuretic hormone (AVP) secretion, regulated by extracellular fluid solute concentration, blood volume, cardiac filling pressure, and arterial pressure, is heightened in HF due to sympathetic and renin-angiotensin-aldosterone system activation, particularly during acute water overload. Elevated serum osmolality may intensify AVP release, with AVP adversely affecting cardiovascular dynamics and promoting HF progression (19, 20). Secondly, increased serum osmolality may disrupt venous reserve distribution. High arterial and non-visceral venous osmolality can shift fluid from venous reserves to the effective circulation, raising preload and leading to congestion. This may manifest as elevated pulmonary artery pressures and potentially exacerbate pulmonary edema, evidenced by a link between osmolality and increased extravascular lung water. Finally, elevated osmolality could impair renal function through several mechanisms. Heightened AVP levels may aggravate chronic kidney disease, while high serum osmolality may induce renal tubular injury and fibrosis via the sympathetic nervous system and aldose reductase pathway, culminating in oxidative stress (21, 22). Moreover, increased extracellular osmolality may prompt water excretion, resulting in cellular shrinkage and intracellular dehydration, precipitating local and systemic conditions detrimental to cellular integrity.

In summary, serum osmolality levels serve as a cost-effective and widely accessible metric to assess HF severity and prognosticate mortality risk. This study delineates the association between serum osmolality and 28-days all-cause mortality post-hospitalization, offering a clinical reference point based on serum osmolality levels. The significance of our research is manifold: (1) It elucidates a curvilinear relationship between serum osmolality and all-cause mortality within 28-days all-cause mortality in HF patients under intensive care; (2) It advances the development of short-term prognostic models based on serum osmolality levels for HF patients; (3) It distinguishes higher serum osmolality as an independent determinant of 28-days all-cause mortality, accounting for confounding variables,a contrast to prior studies. (4) The results have been substantiated through Kaplan-Meier survival curve analysis, enhancing the robustness of future research findings; (5)A threshold effect model was utilized to pinpoint the turning point where serum osmolality begins to significantly impact 28-days all-cause mortality; (6) Ethical integrity was maintained throughout the study, with no patients being harmed or ethical boundaries transgressed.

Our study is subject to several limitations: (1) It is confined to examining the relationship between initial serum osmolality and short-term mortality post-hospital admission, without accounting for the potential influence of post-admission medications, such as renin-angiotensin-aldosterone system (RAAS) inhibitors and diuretics, on serum sodium levels and, consequently, serum osmolality; (2) Serum osmolality was estimated using a formula rather than direct measurement, precluding the detection of “delta osmolality” or “osmolal gap”. Although the formula was chosen with diligence, it may not accurately reflect true osmolality values; (3) The study relies on data from the MIMIC-IV database, which predominantly comprises patients with severe HFrEF, thus constraining the broader applicability of the findings; (4) The causal mechanisms linking serum osmolality with 28-days all-cause mortality were not explored, indicating the need for further investigative research.

## Conclusion

Our findings reveal a U-shaped correlation between serum osmolality levels and 28-days all-cause mortality in HFrEF patients post-hospital admission, after accounting for confounding factors. This study enhances the understanding of serum osmolality’s relevance in HFrEF cases. Given its simplicity, ease, and cost-effectiveness, serum osmolality measurement underscores the significance of osmolality management in aiding physicians to identify patients at high risk. Nonetheless, additional research is essential to ascertain whether interventions aimed at serum osmolality levels can favorably alter clinical outcomes in this cohort.

## Data availability statement

The raw data supporting the conclusions of this article will be made available by the authors, without undue reservation.

## Author contributions

QZ: Investigation, Methodology, Visualization, Writing – original draft, Writing – review & editing. JL: Data curation, Writing – review & editing. PL: Data curation, Software, Writing – review & editing. JM: Software, Writing – review & editing. ZW: Conceptualization, Data curation, Writing – review & editing. TT: Conceptualization, Writing – review & editing. GH: Investigation, Writing – review & editing. SS: Conceptualization, Funding acquisition, Investigation, Methodology, Project administration, Software, Supervision, Writing – original draft, Writing – review & editing.
